# Fibroblast Activation Protein Overexpression and Clinical Implications in Solid Tumors: A Meta-Analysis

**DOI:** 10.1371/journal.pone.0116683

**Published:** 2015-03-16

**Authors:** Fang Liu, Li Qi, Bao Liu, Jie Liu, Hua Zhang, DeHai Che, JingYan Cao, Jing Shen, JianXiong Geng, Yi Bi, LieGuang Ye, Bo Pan, Yan Yu

**Affiliations:** 1 Department of Internal Medical Oncology, The Third Affiliated Hospital of Harbin Medical University, Harbin, China; 2 Department of Radiation Oncology, The Second Affiliated Hospital of Harbin Medical University, Harbin, China; 3 Digestion Department of Internal Medicine, General Hospital of Hegang Mining Group, Hegang, Heilonhjiang Province, China; 4 Emergency Department, Heilongjiang Provincial Electric Power Hospital, Harbin, China; University Hospital Llandough, UNITED KINGDOM

## Abstract

**Objective:**

Fibroblast activation protein (FAP) plays a vital role in tumor invasion and metastasis. Previous studies have reported its prognostic value in different tumors. However, the results of these reports remain controversial. In this study, a meta-analysis was performed to clarify this issue.

**Methods:**

A search of the PubMed, Embase and CNKI databases was conducted to analyze relevant articles. The outcomes included the relations between FAP expression and histological differentiation, tumor invasion, lymph node metastasis, distant metastasis and overall survival (OS). Sensitivity analysis by FAP expression in different cells and tumor types were further subjected to sensitivity analyses as subgroups. Pooled odds ratios (ORs) and hazard ratios (HRs) were evaluated using the random-effects model.

**Results:**

The global analysis included 15 studies concerning various solid tumors. For global analysis, FAP overexpression in tumor tissue displayed significant associations with poor OS and tumor progression (OS: HR = 2.18, *P* = 0.004; tumor invasion: OR = 4.48, *P* = 0.007; and lymph node metastasis: OR = 3.80, *P* = 0.004). The subgroup analyses yielded two notable results. First, the relation between FAP overexpression and poor OS and tumor lymph node metastasis was closer in the patients with FAP expression in tumor cells. Second, the pooled analyses of colorectal cancers or pancreatic cancers all indicated that FAP overexpression was associated with a detrimental OS (HR: 1.72, *P* = 0.009; HR: 3.18, *P* = 0.005, respectively). The magnitude of this effect was not statistically significant compared with that in patients with non-colorectal cancers or non-pancreatic cancers. These analyses did not display a statistically significant correlation between FAP expression and histological differentiation and distant metastasis in all of the groups.

**Conclusions:**

FAP expression is associated with worse prognosis in solid tumors, and this association is particularly pronounced if FAP overexpression is found in the tumor cells rather than the stroma.

## Introduction

In recent years, the close correlation between cancer and its microenvironment regarding tumor growth, invasion and metastasis has become increasingly apparent [1–3]. Strong experimental evidence has shown that stromal fibroblasts, which are an essential component of the tumor microenvironment and which have often been designated as cancer-associated fibroblasts (CAFs), can promote tumorigenesis and progression through multiple mechanisms, including proliferation, angiogenesis, invasion, survival and immune suppression [4–6]. Reports continue to accumulate evidence suggesting that FAP, which is an important marker for CAFs, plays a predominant role in the progression of many tumor types [7]. FAP is expressed in reactive CAFs in stroma and granulation tissue to promote wound healing. Recently, some studies have reported FAP expression in some cancerous epithelial cells and osteosarcoma tumor cells [8–11]; however, FAP expression was absent in normal adult tissues [12–14]. Thus, the quantity of FAP most likely presents an important prognosis for the clinical behavior of tumors. However, some earlier studies addressing the latter notion provided no consistent conclusion based on single-patient cohorts or on explorative evaluations. Some studies have analyzed both the intensity and proportion of FAP expression by immunohistochemistry (IHC); these studies suggested that stromal FAP expression promoted tumor prognosis and poor survival in some solid tumor types, including colon cancer [15] and pancreatic adenocarcinoma [16]. However, another study concerning colorectal cancer did not obtain statistical significance regarding FAP expression [17]. In 2001, Naohiro et al observed statistical significance regarding FAP expression in breast cancer [18]. Therefore, these results require further exploration to validate FAP expression as a novel prognostic marker and therapeutic target in cancer. In the current study, a comprehensive approach was used, and a meta-analysis was performed to assess the effect of FAP expression on survival and clinicopathological characteristics in solid tumors.

## Materials and Methods

### Search strategy and study selection

The literature in this study was found according to preferred reporting items for meta-analyses statements[19]. The PubMed, Embase and China National Knowledge Infrastructure (CNKI) databases were searched, covering all studies reported through April 2014 with the following terms: “fibroblast activation protein or cancer-associated fibroblasts or FAP or CAF” and “cancer or tumor or malignancy” and “outcome or prognosis or survival or response or efficacy”. Reviews and other relevant articles were searched to identify all potential results. The citation lists of the retrieved articles were manually screened to ensure the efficiency of the search strategy.

**Fig 1:**
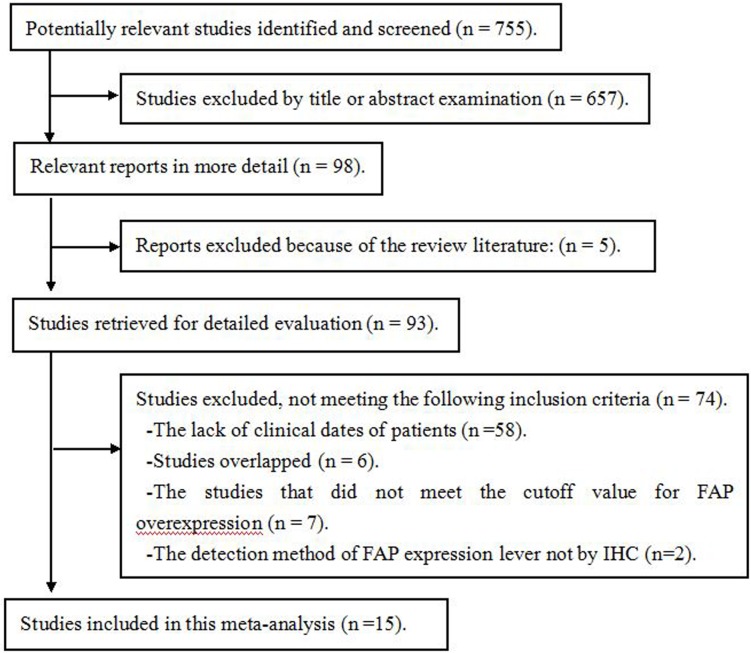
Flow chart of study inclusion.

The included articles were published in journals and provided the outcome data of FAP expression and the clinical information of patients. The process of selecting publications is shown in Fig. 1. The eligibility criteria were the detection of FAP expression levels by IHC, with cutoff values of FAP overexpression, the availability of primary interest outcomes, odds ratio or hazard ratio values for five of the above-mentioned objectives and publications in the English or Chinese language. The exclusion criteria were as follows: (1) non-normal control studies compared with FAP overexpression; (2) control information that was missing or that could not be acquired by our repeated requests or calculations; (3) studies that did not examine solid tumors; and (4) reviews and letter articles.

### Data extraction

Two investigators (Fang Liu and Li Qi) independently screened the articles and extracted the data from the selected studies using standard data-abstraction forms. Any disagreements were resolved through discussion with another reviewer (Yan Yu). When the original data could not be found in the published papers, we contacted the authors using the email address provided in the articles.

The following information was collected from each study: the name of the first author, year of publication, country of origin, tumor type, number of patients, clinical stage, presence or absence of adjuvant therapy, treatment outcome, FAP detection method, IHC antibody, FAP location by IHC staining, FAP-positive case number, cutoff for overexpression, primer case numbers classified according to the cutoff value, ORs with their 95% confidence intervals (95% CIs) for clinicopathological characteristics mentioned previously, and HRs for OS with their 95% CIs. If HRs and 95% CIs were not available directly from a report, then the methods described by Tierney et al [20] to obtain an indirect estimated value were used. The survival rates were read by the Engauge Digitizer version 4.1 software from Kaplan-Meier curves, and then the data were entered in the spreadsheet appended to the article by Tierney et al for calculation [21].

### Coding of categorical variables

Because different scales of FAP IHC staining were observed across the papers, the following rules were applied: (1) when the expression level was encoded as high/low, then no change was applied; (2) for the percentage data of semi-quantitative scoring, staining > 10% (++) was regarded as high expression and vice versa; (3) when the data were encoded by zero, one, two, and three according to the intensity of FAP staining because of the presence of > two categories as grading/staging scales, then stages two and three and stages zero and one were grouped as high expression and low expression, respectively.

For histological differentiation, the following category rules were applied: (1) when the histological grade was encoded as high-moderate and moderate-poor, then no change was applied; (2) when the grade was encoded by G1, G2 and G3, according to grading scales G1 and G2, G3 was divided accordingly into high grade and low grade; (3) when the grade was encoded by well differentiated, moderately differentiated and poorly differentiated, then well differentiated and moderately differentiated, and poorly differentiated were grouped accordingly as high grade and low grade, respectively.

For the state of tumor invasion, when the invasion state was encoded “with/no”, then no change was applied; otherwise, stage T3/4 was considered local tumor invasion, and stage T1/2 was considered no local tumor invasion.

### Statistical methods

The meta-analysis was implemented using the STATA 11.0 software. The pooled ORs were computed to evaluate the magnitude of the association between FAP overexpression and poor histological differentiation, tumor invasion, lymph node metastases, and distant metastases. The pooled HRs were calculated to assess the magnitude of the correlation between FAP overexpression and OS. Next, these ORs and HRs were weighted and pooled across studies using corresponding models. The effect of heterogeneity was measured using Higgins *I* ^2^ statistic [22]. A random-effects model (DerSimonian & Laird) was used for meta-analysis when the result of the Q-test (*P* < 0.05 or *I* ^*2*^ > 50%) claimed heterogeneity among the studies. Otherwise, the fixed-effects model (Mantel and Haenszel) was used.

To decrease some of the outcome effects of heterogeneity among the studies, subgroup analyses were conducted based on two facts. One analysis was based on the presence or absence of FAP overexpression in tumor cells. Group B included patients with FAP expression in tumor cells and in or not in tumor stroma [8–11]. Group A included patients with FAP expression in tumor stroma but not in tumor cells [15–18,23–25,27–30]. The differences between group B and group A were compared. The other subgroup analysis was based on each type of cancer, and then that information was compared with other types of cancer. The groups were the colorectal cancer group, pancreatic cancer group and other cancer group.

The stability of the pooled results was confirmed by sensitivity analyses. Publication bias was statistically assessed by Begger’s test (*P* < 0.05 indicated significant publication bias) and depicted by funnel plots.

## Results

### Description of studies

Fifteen studies that used IHC techniques for the assessment of FAP expression levels and location were identified. In total, 1,998 patients in seven states were included in our meta-analysis. The clinical characteristics of the included studies are listed in Table 1. The analysis included eleven solid tumor types: colorectal cancer, pancreatic adenocarcinoma, non-small cell lung cancer, esophageal cancer, gastric cancer, ovarian carcinoma, breast cancer, medullary thyroid carcinomas, endometrial carcinoma, oral squamous cell carcinoma, and osteosarcoma. In total, 1,914 patients (95.80%) were not treated before surgery. Sixty-six and 18 patients underwent neoadjuvant chemotherapy and preoperative irradiation, respectively. The laboratory information from the selected studies is presented in Table 2. Tissue specimensapp:ds:specimen from 13 studies were obtained from surgical procedures, and the specimens from two studies were collected at the Department of Pathology [28, 30].

**Table 1:** Studies and clinical information of patients included in this meta-analysis.

Study	Year	Country	Disease	Case (n)	Stage	Adjuvant therapy before surgery	Outcome provided
Paulette Mhawech-Fauceglia et al (11)	2013	USA	Epithelial ovarian carcinoma	66	advanced	Neoadjuvant chemotherapy	OS/distant metastases
Dong Tang Yuan et al (9)	2013	China	Osteosarcoma	160	IIA-III	No	OS/histological differentiation/tumor invasion/lymph node metastasis/distant metastases
Min Shi et al (8)	2012	China	Pancreatic adenocarcinoma	134	I-IV	No	OS/histological differentiation/tumor invasion / lymph node metastasis/distant metastases
Mariusz Adam Goscinski et al (10)	2008	Norway	Esophageal adenocarcinoma	69	I-IV	18 preoperative irradiation/51 no	Tumor invasion/distant metastases
Maria L et al (24)	2013	Sweden	Colorectal cancer	449	I-IV	No	OS/histological differentiation/distant metastases
Mercedes Herrera et al (17)	2013	Spain	Colorectal cancer	289	I-IV	No	OS
Yida Liao et al (23)	2013	China	Non-small cell lung cancer	59	I-III	No	OS/lymph node metastasis
Rui Fen Wang et al (25)	2013	China	Gastric cancer	60	I-IV	No	Histological differentiation/tumor invasion /lymph node metastasis/distant metastases
Jing Song et al (27)	2011	China	Endometrial carcinoma	216	I-III	No	Histological differentiation/tumor invasion /lymph node metastasis
Steven J et al (16)	2008	USA	Pancreatic adenocarcinoma	70	I-III	No	OS/lymph node metastasis
Oskar Koperek et al (28)	2007	Austria	Medullary thyroid carcinomas	28	pT1-4	No	Tumor invasion
Leonard R et al (15)	2007	Sweden	Colorectal cancer	138	I-IV	No	OS/histological differentiation/distant metastases
Naohiro Ariga et al (18)	2001	Japan	Invasive ductal carcinoma of the breast	112	I-III	No	OS
Ling Zhang et al (29)	2008	China	Oral squamous cell carcinoma	80	I-III	No	Histological differentiation/lymph node metastasis
Hai Yun Wang et al (30)	2009	China	Esophageal cancer	68	I-IV	No	Histological differentiation/tumor invasion/lymph node metastasis

Abbreviations: OS, overall survival.

**Table 2:** Evaluation and outcomes of FAP expression by IHC in the selected studies.

Study	FAP detection method	FAP location by IHC stain	Positive number(%)	FAP antibody	Cut-off for overexpression
Paulette Mhawech-Faucegliaet al (11)	IHC	Stroma^+^ tumor^+/−^	86.4	Polyclonal antibody to FAP-alpha, Imgenex, San Diego, CA, USA.	The intensity as (−) for negative expression, (1+) for weak expression, (2+) for moderate expression and (3+) for strong expression. The score of two assessments was reviewed, and when a discrepancy in scoring existed, a consensus was reached. Outcomes were negative (−) and positive (+).
		Tumor^+^ stroma^+-^	50.0		
Dong TangYuan et al (9)	IHC	Tumor cells	100	Rabbit polyclonal, ab53066, Abcam, Hong Kong Ltd.	The percentage scoring: 0 (0%), 1 (1–10%), 2 (11–50%) and 3 (> 50%). The staining intensity scoring: 0 (negative), 1 (weak), 2 (moderate) and 3 (strong). An IRS was obtained for each case by multiplying these two scores. The IRS median cutoff value was 4.68.
Min Shiet al (8)	IHC	Stroma	73.1	Rabbit anti-human polyclonal antibody, LifeSpan BioSciences Inc, USA; dilution: 1:70.	Staining area scoring: 0 (≤ 10%), 1 (> 11% to ≤ 25%), 2 (> 26% to ≤ 50%) and 3 (> 51%). The method of staining intensity scoring differed from that by DongTang Yuan et al. The sum of the two scores was the final score. A final score ≥ 3 indicated positive expression.
		Tumor cells	76.1		
Mariusz Adam Goscinski et al (10)	IHC	Stroma	89.9	ab53066, isotype IgG, Abcam Cambridge, UK; dilution: 1:100.	The percentage scoring: 0 = 0 positive cells, 1 ≤ 25%, 2 = 25–49%, and 3 ≥ 50% positive cells. The method of staining intensity scoring was different from that by Dong Tang Yuan et al. The outcome was achieved by multiplying the corresponding with percentage scoring and intensity scoring and was divided into four final groups: 0, 1 +, 2+ and 3 +. High expression: cases with 2+ and 3 +.
		Cancer cells	98.6		
Maria L et al (24)	IHC	Stroma	90.0	Monoclonal antibody D8; Vitatex, Stony Brook, NY, USA; dilution: 1:100.	Stromal staining was assessed as negative, +, ++ and +++ according to the semiquantitative scale suggested by Leonard R et al (15).
Mercedes Herreraet al (17)	IHC	Stroma	72.0	Polyclonal antibody to FAP-alpha; Imgenex, San Diego, CA, USA.	IHC data were evaluated as “low” or “high” expression regarding the rate of positive cells for each sample and each marker.
Yida Liaoet al (23)	IHC	Stroma	76.2	Rabbit polyclonal human antibody, Abcam, UK.FAP-a, ab53066, Abcam; diluted 1:200.	In each section, eight random fields were picked to assess the expression levels of FAP-a. Next, an average score was calculated. The intensity and percent or stroma staining were evaluated as suggested by Leonard R et al (15).
Rui Fen Wanget al (25)	IHC	Stroma	100	FAP primary monoclonal antibody, 2 μg/mL, R&D Systems, Minneapolis, MN.	The staining in cancer stroma was classified into three groups: +++, strong staining in > 50% of stroma fibroblasts; ++, moderate staining in > 50% of stroma fibroblasts; and +, faint or weak staining in > 50% of stroma fibroblasts. +++ as the high-expression group; ++ and + as the low-expression group.
Jing Songet al (27)	IHC	Stroma	89.9	Rabbit anti-human FAP polyclonal antibody, Abcam, UK.	The stroma cutoff for the overexpression method was similar to that by Min Shi et al.
		Tumor cells	rare		
Steven Jet al (16)	IHC	Stroma	90.0	D8, FAP/seprase antibody, SUNY, Stony Brook, NY, USA.	FAP staining was graded as suggested by Leonard R et al (15) according to the semiquantitative scale.
		Tumor cells	rare		
Oskar Kopereket al (28)	IHC	Peritumoral Stroma	92.9	F19, mouse host; dilution 1:20; Garin-Chesa.	According to the semiquantitative scale immunoreactivity was graded as follows: negative (-), weak (+), moderate (++) and strong (+++). (+ +) and (+++) were the high-expression group.
		Intratumoral Stroma	78.6		
Leonard Ret al (15)	IHC	Stroma	93.0	Rabbit anti-human FAP monoclonal antibody D8.	Using semiquantitative analysis, stromal staining was different from that by Dong Tang Yuan et al. Groups scored with 0 or 1 staining were compared with those with greater (2 or 3) staining.
NaohiroArigaet al (18)	IHC	Stroma	54.0	Using all MAbs (D8, D28, D43 and F19).	Expression in the stromal area was semiquantitatively analyzed using the same method as Leonard R et al (15). Cases were classified as (-) and (+) for scanty expression. (+) and (++) were classified as abundant expression.
Ling Zhanget al (29)	IHC	Stroma	65.0	Anti-human rat neomarkers, Abnoval.	Each section (×200) had five horizons in tumor concentration areas. An FAP positive cell percentage was calculated in a horizon: > 5% was positive; < 5% was negative.
Hai Yun Wang et al (30)	IHC	Stroma	83.0	Rabbit anti-human FAP polyclonal antibody, Abcam, UK.	From each section, five horizons were chosen in the tumor concentration areas, and the FAP positive cell percentage was calculated in a horizons. > 10% was positive; < 10% was negative. Five horizons per section was the average number.

Abbreviations: FAP, fibroblast activation protein; IHC, immunohistochemistry.

**Fig 2:**
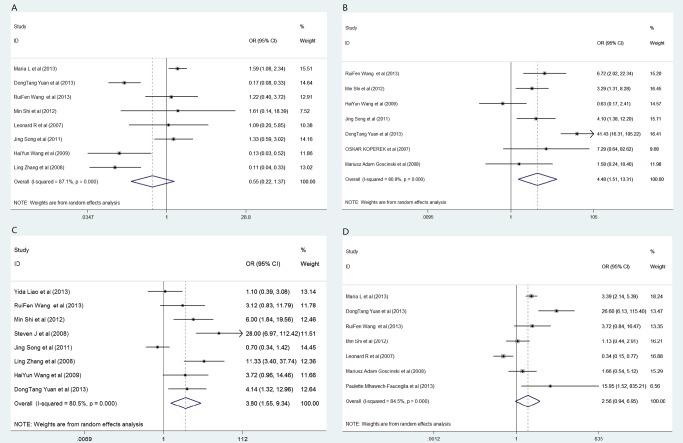
Forest plot of clinicopathological characteristics and FAP expression in patients with solid tumors. (A) histological differentiation; (B) tumor invasion; (C) lymph node metastasis; (D) distant metastases. OR, odds ratio; 95% CI, 95% confidence interval.

The IHC method was used to detect FAP expression in the tumor tissues of patients. Descriptions of the antibodies used in the included studies are provided in Table 2. Staining with various antibodies was used to evaluate the FAP expression levels and expression status in tumor cells and/or in stroma, although four studies used antibody ab53066 [9,7,10,23], and another four studies used antibody D8 [15,16,18,24]. Some studies used a combined evaluation of cytoplasmic and membrane staining for determining the FAP expression status. The cutoff for overexpression depended on the staining score and on the method used. Because of the high test results of the total heterogeneity in all category analyses (*I* ^2^ > 50%, *P* > 0.05, Fig. 2), the random-effects model was used to assess the correlation between FAP expression and survival and clinicopathological characteristics.

### Histological differentiation

**Fig 3:**
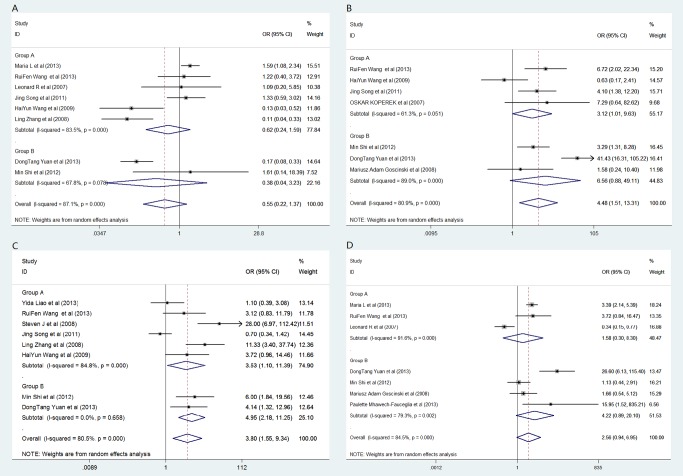
Forest plot of clinicopathological characteristics and FAP expression in solid tumors by the stratification analysis based on FAP expression cell. (A) histological differentiation; (B) tumor invasion; (C) lymph node metastasis; (D) distant metastases. Group B includes patients with FAP expression in tumor cells and in or not in tumor stroma. Group A includes patients with FAP expression in tumor stroma but not in tumor cells. Abbreviations: OR, odds ratio; HR, hazard ratio; 95% CI, 95% confidence interval.

**Fig 4:**
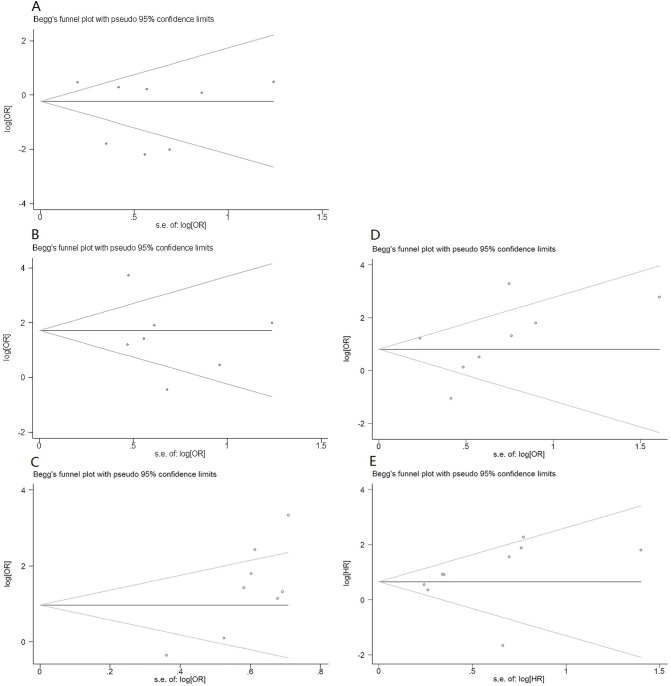
Begger’s funnel plot for trials comparing the effect of FAP expression in solid tumors on (A) poor histological differentiation; (B) tumor invasion; (C) lymph node metastasis; (D) distant metastases; (E) overall survival. Abbreviations: OR, odds ratio; HR, hazard ratio.

Eight studies that included 1,305 patients could be used to analyze the relation between FAP expression and the histological differentiation of the solid tumors (Table 3). The patient-pooled analysis revealed that the FAP expression level did not significantly correlate with tumor histological differentiation (OR: 0.55, 95% CI: 0.22–1.37, *P* = 0.197; Fig. 2A). Further stratificationapp:addword:stratification analysis revealed no observable correlation in group A (OR: 0.62, *P* = 0.323) or in group B (OR: 0.38, *P* = 0.375; Fig. 3A). The funnel plot revealed that the statistical results did not demonstrate publication bias (*P*_Begg_ = 0.805 for all patients; Fig. 4A; Table 3).

**Table 3:** Association between FAP expression and the clinical characteristics of tumors.

	**Histological differentiation**				**Depth of tumor invasion**			
	**Study** *	**Patient**	**OR(95% CI)**	***P***	**Study** *	**Patient**	**OR(95% CI)**	***P***
**All studies**	8	1305	0.55 (0.22–1.37)	0.197	7	735	4.48 (1.51–13.31)	0.007
**Group A**	6	1011	0.62 (0.24–1.59)	0.323	4	372	3.12 (1.01–9.63)	0.048
**Group B**	2	294	0.38 (0.04–3.23)	0.375	3	363	6.56 (0.88–49.11)	0.067
**Deviations**				0.805^#^				0.652^#^
	**Lymph node metastasis**				**Distant Metastases**			
	**Study** *	**Patient**	**OR(95% CI)**	***P***	**Study** *	**Patient**	**OR(95% CI)**	***P***
**All studies**	8	847	3.80 (1.55–9.34)	0.004	7	1076	2.56 (0.94–6.95)	0.065
**Group A**	6	553	3.53 (1.10–11.39)	0.035	3	647	1.58 (0.30–8.30)	0.588
**Group B**	2	292	4.95 (2.18–11.25)	< 0.001	4	429	4.22 (0.89–20.10)	0.070
**Deviations**				0.048				0.293^#^

Abbreviations: OR, odds ratio; HR, hazard ratio; CI: confidence interval.

*Study: The number of studies included in the analysis.

# *P* > 0.05 indicates no publication bias.

### State of tumor invasion

Seven trials that included 735 subjects were eligible for the final analysis. Four trials were included in group A, and three were included in group B (Table 3). The pooled outcome from all patients indicated that the patients with high FAP expression had a higher ratio of local tumor invasion than those patients with low FAP expression (OR: 4.48, *P* = 0.007; Fig. 2B). In addition, the stratified analysis, which located FAP-expressing cells, demonstrated a similar correlation in group A (OR: 3.12, *P* = 0.048; Fig. 3B). Nevertheless, in group B, the statistical outcomes revealed that patients with FAP overexpression in tumor cells had a greater risk of local tumor invasion than those patients with FAP overexpression in stroma cells in group A (OR: 6.56). However, this difference was not statistically significant (95% CI: 0.88–49.1, *P* = 0.067). No publication bias was observed, as determined by the funnel plot (*P*_Begg_ = 0.652 for all patients; Fig. 4B; Table 3).

### Lymph node metastases

Eight trials that included 847 patients assessed lymph node metastasis. High FAP expression in all patients significantly increased the risk of lymph node metastasis (OR: 3.80, *P* = 0.004; Fig. 2C). Further stratification analysis indicated that the patients with FAP overexpression in group B had a greater risk of lymph node metastasis than those patients in group A (OR: 4.95, *P* < 0.001 vs OR: 3.53, *P* = 0.035, respectively; Fig. 3C; Table 3). The statistical results indicated signs of publication bias, as determined by the funnel plot (*P*_Begg_ = 0.048 for all patients; Fig. 4C; Table 3).

### Distant metastases

Data from seven studies that included 1,076 patients were applicable for distant metastasis analysis and subgroup analysis. As shown in Figs. 2D and 3D, high FAP expression significantly increased the risk of distant metastases in all patients (OR: 2.56). This risk was predominantly enhanced in group B (OR: 4.22), with no statistically significant difference (CI_all patients_: 0.94–6.95, *P*_all patients_ = 0.065; CI_group B_: 0.89–20.10, *P*_group B_ = 0.07; Table 3). Nevertheless, in group A, which included three studies with 647 patients, FAP expression did not correlate with distant metastases of the tumors (OR = 1.58, CI: 0.30–8.30, *P* = 0.588). The funnel plot indicated no publication bias (*P*_Begg_ = 0.293 for all eight patients included in the studies; Fig. 4D; Table 3).

### Overall survival

**Fig 5:**
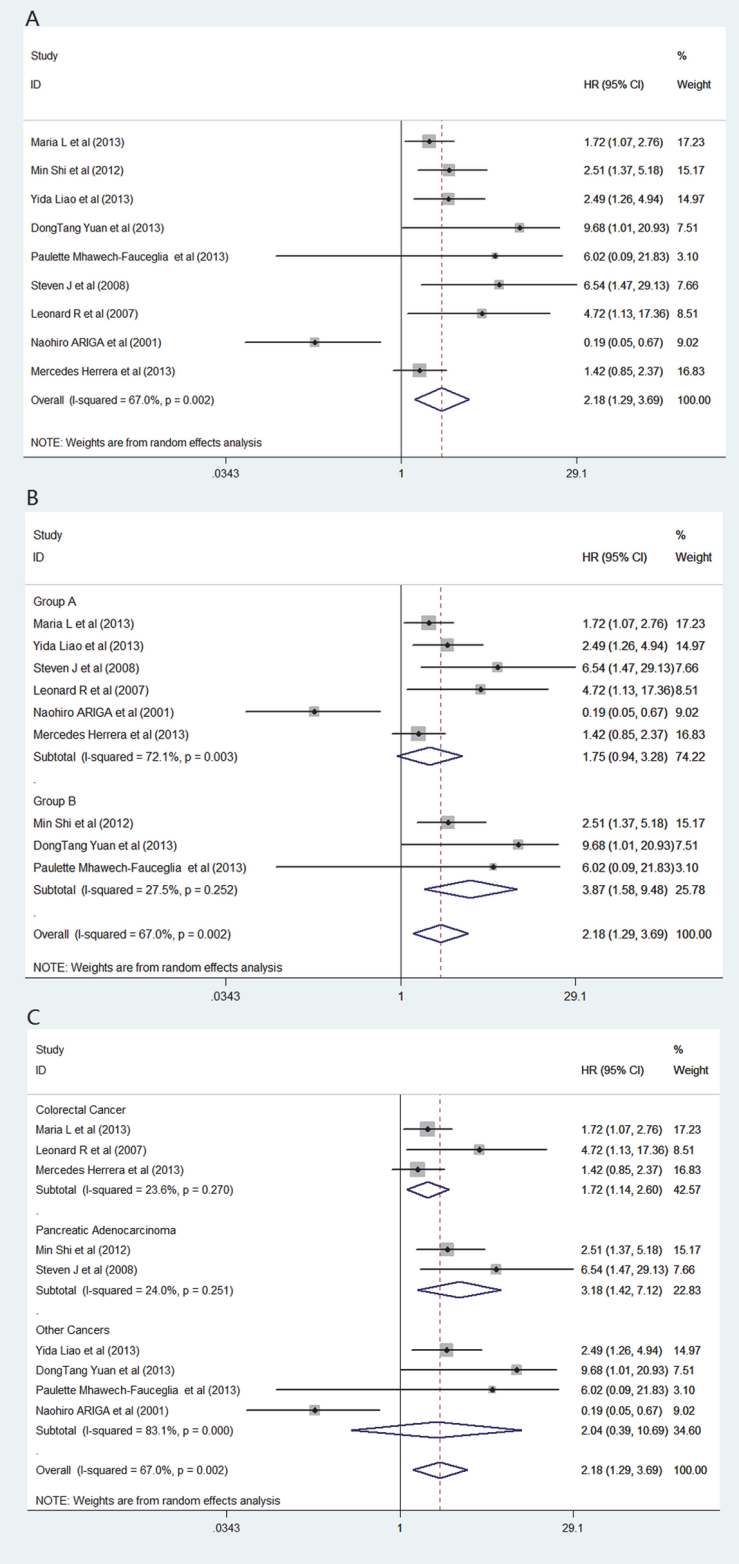
Forest plot of overall survival and FAP expression in solid tumors. (A) Forest plot of pooled total studies; (B) subgroup analysis by FAP expression status in different cells; (C) subgroup analysis by tumor type.

Nine studies that included 1,490 patients were eligible for the analysis. As shown in Fig. 5A, the pooled outcome for all patients indicated a significant correlation between the patients with FAP overexpression and poor OS (HR = 2.18, CI: 1.29–3.69, *P* = 0.588). The stratified analysis according to FAP expression status demonstrated a closer correlation to the patients with FAP overexpression in tumor cells (HR_group B_: 3.87, CI: 1.58–9.48). Additionally, a statistically significant difference was observed for the patients in group A (*P* = 0.004; Table 4). Nevertheless, in group A, the statistical outcomes revealed that patients with FAP overexpression only in stroma tumors had a tendency of poor survival (HR: 1.75, CI: 0.94–3.28) and that this effect was not statistically significant (*P* = 0.08).

**Table 4:** The association between FAP expression and the overall survival of patients with solid tumors.

	Study*	Patient	HR(95% CI)	P	Subgroup difference P	Deviations(p)
**All studies**	9	1490	2.18(1.29–3.69)	0.004		0.052#
**Group A**	6	1126	1.75(0.94–3.28)	0.08		
**Group B**	3	364	3.87(1.58–9.48)	0.003		
**Colorectal Cancer**	3	876	1.72(1.14–2.60)	0.009	0.699	
**Pancreatic Cancer**	2	204	3.18(1.42–7.12)	0.005	0.864	
**Others Cancers**	4	410	2.04(0.39–10.69)	0.397		

Abbreviations: HR, hazard ratio; CI: confidence interval.

*Study: The number of studies included in the analysis.

# *P* > 0.05 means no publication bias.

In addition, sensitivity analysis by tumor type indicated that an association between FAP overexpression and poor survival was evident for the three colorectal studies (HR: 1.72, 95% CI: 1.58–9.48, *P* = 0.009) (Fig. 5C; Table 4). This deviation from the pooled estimate was not statistically different from that of non-colorectal cancers (subgroup difference *P* = 0.699). Compared with low FAP expression, the pooled analyses of the two pancreatic cancer studies indicated that FAP overexpression was associated with a greater detrimental outcome (HR: 3.18, 95% CI: 1.42–7.12, *P* = 0.005). The magnitude of this effect was not statistically significant compared with that in non-pancreatic cancers (subgroup difference: *P* = 0. 864) (Fig. 5C; Table 4). The funnel plot indicated no significant publication bias (*P*_Begg_ = 0.095; Fig. 4E; Table 4).

## Discussion

In the present global meta-analysis of patients with solid tumors, FAP javascript:void(0);overexpression displayed a significant association with poor overall survival, which was linked to tumor progression risks, causing increased odds ratios of tumor invasion and lymph node metastasis. Further subgroup analysis based on the FAP expression status in different cells revealed that the patients with FAP overexpression in tumor cells had a higher risk of poor overall survival and greater odds ratios of lymph node metastases. In those patients with FAP expression only in stroma tumors, although tumor invasion and lymph node metastasis correlated with FAP overexpression, the overall survival of these patients was not statistically significant compared with that in patients with lower FAP expression. The patients with FAP overexpression (particularly expression in tumor cells) tended to have a higher risk of distant metastases; however, this risk was not significantly different from those of the other groups. Nevertheless, the pooled statistical result demonstrated that FAP expression had no significant correlation with the histological differentiation of the tumors in any group.

In addition, although colorectal cancer or pancreatic cancer was associated with FAP overexpression and poor survival, sensitivity analysis by tumor type indicated that the effect was not statistically significant compared with that in non-colorectal cancers or non-pancreatic cancers. Thus, the association between FAP overexpression and other tumor types was not evident.

Previous studies have demonstrated that cancer tissues are composed not only of cancer cells but also of cancer-associated stromal cells, including fibroblasts, extracellular matrix molecules, endothelial cells and immune cells [11, 26]. Moreover, the occurrence and development of tumors are not only determined unilaterally by epithelial or mesenchymal cells but also by the equilibrium state created by the interaction of a tumor and the host interface of a tumor’s microenvironment [31, 32]. Therefore, in recent years, increased attention has been given to the host interface of a tumor’s microenvironment, which was considered as important as cancer cells themselves in the progression and metastasis of tumors. Based on strong experimental evidence, CAFs, which are essential components of the tumor microenvironment, were shown to play an important role in both tumor progression and the regulation of the tumor microenvironment by the secretion of soluble factors, such as FAP and extracellular matrix modifiers [33]. FAP, which is a membrane serine protease, is considered an important marker of activated CAFs during tissue remodeling [34]. In addition, FAP expression can be induced in non-transformed, activated stromal fibroblasts but is not expressed in fibroblasts in normal tissues [35]. Particularly in human malignancies, FAP expression is often detected on the surface of fibroblasts surrounding epithelial cancers, including pancreatic cancer [36], colon cancer [17], prostate cancer [37], breast cancer [38] and skin cancer [39], as well as in some bone sarcomas and soft tissues [40]. Notably, some reports have observed FAP overexpression in both cancer cells and adjacent stroma [41–43]. Functionally, FAP can enhance stromal cell proliferation and invasiveness and affect cell apoptosis primarily when this protein correlates with increased tumorigenicity due to the proteolysis of the extracellular cell matrix (ECM) [9,10]. The cancer-specific distribution and function of FAP make this protein eligible as a novel prognostic marker and therapeutic target in tumors. Previously, several studies reported the relation between FAP overexpression and the clinical characteristics and outcome in solid tumor patients. Unfortunately, the results differed among various studies, and no consensus has been reached. Moreover, prior research regarding small-molecule inhibition of FAP was not as effective as expected [44]. However, recent studies targeting FAP using a novel FAP-activated prodrug that alters the activation of a cytotoxic compound in the tumor stroma [45] have reported promising results. Therefore, the effect of FAP overexpression on tumor stroma or tumor cells or on different types of tumors requires further assessment.

In this study, positive FAP expression ranged between 50% and 100% (Table 2). FAP was not only consistently expressed in the peritumoral and intratumoral stromal compartment of carcinomas but also in some types of tumor cells, such as pancreatic adenocarcinoma, osteosarcoma, esophageal adenocarcinoma and epithelial ovarian carcinoma [8,10–11]. In osteosarcoma, FAP expression occurred only in tumor cells [9]. FAP expression was detected rarely in some cancer cells, such as differentiated carcinomas [16] and endometrial carcinoma [27]. In other studies, FAP expression was only reported in stroma tumors [15,17–18,23–25,28–30]. Levels of FAP overexpression ranged between 54% and 93% in stroma tumors and between 50% and 100% in tumor cells. In the peritumoral compartment, FAP expression was predominantly found in adjacent CAFs close to tumor cells, with less expression in the surrounding CAFs [8]. In addition, stromal FAP expression was found in endothelial and lymphoid cells in esophageal adenocarcinoma [10]. FAP immunostaining was primarily localized on the cell membrane and in the cytoplasm of tumor cells and CAFs [9], as well as occasionally in the gland lumens [8]. Thus, different cellular and subcellular FAP expression patterns are the foundation of stratification meta-analysis.

These studies revealed several important implications. First, these studies demonstrate that FAP overexpression is associated with a dismal outcome, indicating that this protein may be a promising therapeutic target. This finding is important because the lack of efficacy reported for the small-molecule inhibition of FAP raised doubts regarding the clinical relevance of targeting FAP [44]. However, recent studies targeting FAP using a novel FAP-activated prodrug have reported promising results [45]. Moreover, prior studies addressing FAP expression presented no coherent conclusion based on single-patient cohort evaluations, even with similar tumor types, such as colorectal cancer [15,17]. Second, the studies identified a subgroup of tumors with worse outcome, which is potentially the subgroup in which FAP is overexpressed in tumor cells. Additionally, the studies demonstrated that targeting FAP expression in tumor cells may be a novel therapeutic target. The origin of CAFs has been suggested to be local fibroblasts or bone marrow-derived cells that are recruited into the developing tumor and that adopt a CAF phenotype [46]. These cells originate can also from epithelial or endothelial cells. The epithelial-mesenchymal transition (EMT) is recognized as one potential mechanism of the migration, invasion and metastasis of tumor cells through the transition from epithelial-derived cancer cells to a more mesenchymal-like state [47–49]. Thus, endothelial-mesenchymal conversion may be categorized as a specialized pattern of EMT, which could be the origin of FAP, according to Zeisberg et al [50]. Therefore, prior studies might not have been as effective as expected because these studies did not fully consider cellular localization differences in FAP expression. Further studies regarding FAP inhibition should focus on tumor cells with FAP overexpression instead of only mesenchymal cells. Third, fortunately, the analyses indicate that worse outcomes correlated with a higher risk of lymph node metastasis in patients with FAP overexpression, demonstrating that node metastasis might be a primary approach for tumor migration in these patients. Fourth, the studies not only identified a subgroup of different cancer types but also found that the pooled analyses indicated an association between FAP expression and many-sided clinical implications in solid tumors. Fifth, this study is the first meta-analysis of the effect of FAP expression on the survival and clinicopathological characteristics of patients with solid tumors. Moreover, this study performed innovative subgroup analyses in patients with FAP overexpression in tumor cells or with different types of cancers, and the expected results were obtained. Finally, the analyses emphasized the value of identifying surrogate markers of FAP activation. This study and others [8,15,16,23,24] have suggested that FAP overexpression in tumor, particularly in tumor cells, is a potential key marker.

Nevertheless, several limitations of this meta-analysis should be noted. First, because this study is a literature-based analysis regarding different types of tumors, the potential for publication bias exists because positive results were predominantly published, inflating this study’s final estimate. Second, differences in including tumors affecting different organs, several characteristics of the study designs, and the inclusion of patients whose treatment included adjuvant therapy may have caused wide heterogeneity in the results among the included studies. Stratified analysis of each analysis characteristic corresponding to a tumor type or treatment-related factors would be helpful to reduce the heterogeneity and to improve the quality of the meta-analysis. However, limited studies provided information concerning FAP expression by subgroups; thus, such analyses are impossible. Finally, the role of FAP interactions with the tumor environment [51] was not assessed in this analysis because the original data from the selected studies did not contain such information.

In conclusion, this meta-analysis indicated that patients with FAP overexpression in solid tumors have a higher risk of cancer lymph node metastasis and worse prognosis than patients with low FAP expression. The relationship between FAP and poor prognosis may be stronger in those patients with FAP overexpression in tumor cells relative to patients with FAP overexpression in stroma”. The association between FAP overexpression and a detrimental OS in colorectal cancers and pancreatic cancers is similar to that observed for pooled non-colorectal cancers and non-pancreatic cancers, respectively. This analysis suggests that FAP may be a promising therapeutic approach for developing strategies against this protein, not only aiming at its interstitial expression in the tumor microenvironment but also in tumor cells. Further research must be conducted to ascertain the accuracy of the analysis data from this study regarding tumor types or by further prospective studies with larger sample sizes.

## Supporting Information

S1 PRISMA Checklist PRISMA 2009 Checklist. (DOC)
