# Supplementary material for: Fibroblast Activation Protein Overexpression and Clinical Implications in Solid Tumors: A Meta-Analysis
Source: PLoS One. 2015 Mar 16;10(3):e0116683. doi: 10.1371/journal.pone.0116683 (PMC4361589; doi:10.1371/journal.pone.0116683)
Supplement: S1 PRISMA Checklist — (DOC) [file pone.0116683.s001.doc]

| **Section/topic** | **#** | **Checklist item** | **Reported on page #** |
| --- | --- | --- | --- |
| **TITLE** | | |  |
| Title | 1 | Fibroblast Activation Protein Overexpression and Clinical Implications in Solid Tumors: A Meta-analysis | 1 |
| **ABSTRACT** | | |  |
| Structured summary | 2 | **Background and objective:** In the past years some studies have reported fibroblast activation-protein (FAP) prognostic value in different tumors. however the results of these reports remain controversial. the meta-analysis was performed to clarify this issue. **Data sources:** PubMed, Embase and China National Knowledge Infrastructure (CNKI) databases. **Study eligibility criteria**: the detection of FAP expression levels by IHC or RT-PCR with cutoff values of FAP overexpression, availability of primary interest outcomes, odds ratios or hazard ratios values for six of the above-mentioned objectives and publications in English or Chinese. **Participants** and **interventions:** the tumor [Tissue](app:ds:tissue) [specimens](app:ds:specimens) were [obtain](app:ds:obtain)ed from [surgical operation](app:ds:surgical operation) operations and at the Department of Pathology. **Study Appraisal and synthesis methods:** Data extraction according to FAP expression and location**;** The pooled OR and HRs were calculated to assess the correlation between FAP overexpression and poor [histological](app:ds:histological) [differentiation](app:ds:differentiation), tumor invasion, lymph node metastases, distant metastases, OS and PFS. **Results:** The analysis included 17 studies showed for global analysis, FAP overexpression in tumors tissue had significant associations with poor OS and [tumor](app:ds:tumour) [progression](app:ds:progression). the subgroup analysis indicated that the relation between FAP overexpression and poor OS and tumour progression was closer in groups including patients with FAP expression in tumor cells than in the global analysis. Analysis did not find significant correlation between FAP overexpression and histological differentiation in all groups. **conclusions:** FAP overexpression had a prognostic value for tumor metastsis and a worse prognosis for patients with solid tumors, especially when FAP overexpression was in the tumour cells themselves. **Implications of key findings and limitation:** FAP expression positively correlated with poor survival and high risks of tumour invasions, lymph node metastases and distant metastases, which shows that this protein may be a promising therapeutic target. this is the first meta-analysis on FAP expression's impact on survival and clinicopathological characteristics of patients with solid tumours. Moreover, this study carried out an innovative subgroup analysis in patients with overexpressed FAP in tumour cells. The expected results were obtained and suggests that further study into inhibition of FAP should attache great importance to tumor cells of FAP overexpression instead of only Mesenchymal cells and needs to be conducted to ascertain the accuracy of subgroup analysis according to certain types of tumours or subcellular locations. | 1 |
| **INTRODUCTION** | | |  |
| Rationale | 3 | Cancer-associated fibroblast (CAF) is an essential component of tumor microenvironment. Fibroblast activation-protein( FAP ) as an important mark of CAF plays an vital role in tumor invasion and metastasis . Over the years some studies reported its prognostic value in different tumors. however the results remain controversial.So we performed a meta-analysis to clarify this issue. | 2 |
| Objectives | 4 | (1). To assess the correlation between FAP overexpression and poor [histological](app:ds:histological) [differentiation](app:ds:differentiation), tumor invasion, lymph node metastases, distant metastases. (2). OS. The studies that included patients with FAP overexpression in tumour cells themselves (group B) were further analysed as a subgroup. (3). Pooled odd ratios (ORs) and hazard ratios (HRs) were evaluated. | 2 |
| **METHODS** | | |  |
| Protocol and registration | 5 | NO ( Indicate if a review protocol exists, if and where it can be accessed (e.g., Web address), and, if available, provide registration information including registration number). |  |
| Eligibility criteria | 6 | The detection of FAP expression levels by IHC or RT-PCR with cutoff values of FAP overexpression, availability of primary interest outcomes, odds ratios or hazard ratios values for six of the above-mentioned objectives and publications in english or chinese. | 2 |
| Information sources | 7 | The literature in this study was found according to preferred reporting items for meta-analyses statements. PubMed and CNKI databases were searched, covering all studies reported until April 2014 . | 2 |
| Search | 8 | To search with the following terms: “fibroblast activation protein or cancer-associated fibroblasts or FAP or CAF” and “cancer or tumor or malignancy” and ‘‘outcome or prognosis or survival or response or efficacy’’. | 2 |
| Study selection | 9 | To Screen using keywords, accordinng eligibility. if applicable, included in the meta-analysis (figure 1). | 2 |
| Data collection process | 10 | Two investigators (Fang liu and Li Qi) independently screened the articles and extracted the data from selected studies by using standard data-abstraction forms. Any disagreements were resolved through discussion with another reviewer (Yan Yu). When the original data could not be found in the published papers, we ask the authors by Email Address recorded in articles to provide them. | 3 |
| Data items | 11 | The following information was collected from each study: the name of the first author, the year of publication, the country of origin, the tumor type, the number of patients, the clinical Stage, whether or not there was adjuvant therapy, the outcome, the FAP detection method, the IHC antibody, the FAP location by IHC staining, the FAP Positive Case number, the cutoff for overexpression, the primer case numbers classified according to the cutoff value, the ORs with their 95% confidence intervals (CIs) for clinicopathological characteristics mentioned previously, and HRs for OS and PFS with their 95% CIs. | 3 |
| Risk of bias in individual  studies | 12 | PubMed, Embase and CNKI databases were comprehensively searched, covering all studies reported until April 2014. The included articles were published in journals, providing outcome data of FAP expression and clinical information of patients. Eligible criteria and exclusion criteria were the detection. To extract data Objectively and scientifically. Publication bias was assessed statistically by the Begger's test (*P* < 0.05 indicated significant publication bias) and depicted by funnel plots. | 3 |
| Summary measures | 13 | Using odd ratios (ORs) assessed relation between FAP overexpression and [histological](app:ds:histological) [differentiation](app:ds:differentiation), state of tumor invasion, lymph node metastasis and distant metastasis. Correlation with overall survival (OS) was valued by hazard ratios (HRs) whic is a time relevant risk ratios. | 3 |
| Synthesis of results | 14 | The meta-analysis was implemented using STATA 11.0 software. The pooled ORs and HRs were computed to evaluate the magnitude of the association between FAP overexpression and poor [histological](app:ds:histological) [differentiation](app:ds:differentiation), tumor invasion, lymph node metastases, and distant metastases. Then, these ORs and HRs were weighted and pooled across studies using corresponding models. The effect of heterogeneity was measured by Higgins *I*^2^ statistic. a random-effects model (DerSimonian & Laird ) was used for meta-analysis when the result of Q-test (*P*<0.05 or *I^2^*>50%) claimed heterogeneity among the studies. Otherwise, the fixed-effects model (Mantel and Haenszel) was used. | 3 |

Page 1 of 2

| **Section/topic** | **#** | **Checklist item** | **Reported on page #** |
| --- | --- | --- | --- |
| Risk of bias across studies | 15 | Because this is a literature-based analysis about different kinds of tumors, the potential for publication bias exists because positive results were predominately published, inflating this study's final estimate. Some studies were excluded due to no the detection outcome with cutoff values of FAP overexpression which might be exist the potential publication bias . | 3 |
| Additional analyses | 16 | Subgroup analyses were conducted according to the different locations of overexpression of FAP. In group B, the studies included patients who had FAP overexpression in tumour cells; in stroma tumours, some FAPs were expressed and other FAPs were non-expressed. The others studies were [classified into](app:ds:be classified into) group A. The differences between group B and group A were compared. The stability of pooled results was [confirm](app:ds:confirm)ed by sensitivity analyses. | 3 |
| **RESULTS** | | |  |
| Study selection | 17 | See figure 1. |  |
| Study characteristics | 18 | fifteen studies were identified that used IHC or RT-PCR techniques for the assessment of FAP expression levels and location of expression cells [8-11,15,17-18, 23-30]. A total of 2,297 patients in seven states were included in our meta-analysis. The clinical characteristics of the included studies are listed in Table 1. | 4 |
| Risk of bias within studies | 19 | **Histological differentiation:** The funnel plot revealed that Statistical results did not demonstrate publication bias (*P*_Begg_ > 0.05 for all patients). **State of tumor invasion:** There was no publication bias given from the funnel plot (*P*_Begg_ = 0.652 for all patients). **Lymph node metastases:** The statistical results indicated signs of publication bias according to The funnel plot (*P*_Begg_ = 0.048 for all patients). **Distant metastases:** The funnel plot showed no publication bias (*P* _Begg_ = 0.293 for all eight patients included in the studies). | 4-5.13 |
| Results of individual studies | 20 | See Table 1 and Table 2. | 11-12 |
| Synthesis of results | 21 | The global analysis included 15 studies concerning various solid tumors. For global analysis, FAP overexpression in tumor tissue displayed significant associations with poor OS and [tumor](app:ds:tumour) [progression](app:ds:progression) (OS: HR = 2.18, *P* = 0.004; tumor invasion: OR = 4.48, *P* = 0.007; and lymph node metastasis: OR = 3.80, *P* = 0.004). The subgroup analyses yielded two notable results. First, the relation between FAP overexpression and poor OS and tumor lymph node metastasis was closer in the patients with FAP expression in tumor cells. Second, the pooled analyses of colorectal cancers or pancreatic cancers all indicated that FAP overexpression was associated with a detrimental OS (HR: 1.72, *P* = 0.009; HR: 3.18, *P* = 0.005, respectively). The magnitude of this effect was not statistically significant compared with that in patients with non-colorectal cancers or non-pancreatic cancers. These analyses did not display a statistically significant correlation between FAP expression and histological differentiation and distant metastasis in all of the groups. | 4-5.13 |
| Risk of bias across studies | 22 | Table 3. | 13 |
| Additional analysis | 23 | Further [stratification](app:ds:stratification) [analysis](app:ds:analysis) indicated that the patients with overexpression of FAP in tumor cells had a worse outcome and higher risk of tumour metastasis than those with only stroma expression. The origin of CAFs has been suggested to be local fibroblasts or bone marrow-derived cells that are recruited into the developing tumor and adopt a CAF phenotype. These cells [stem from](app:ds:stem from) epithelial or endothelial cells. Epithelial-mesenchymal transition (EMT) is recognised as one potential mechanism of migration, invasion and metastasis of tumor cells though transition from epithelial-derived cancer cells to a more mesenchymal-like state. Thus, endothelial-mesenchymal conversion may be categorised as a specialised pattern of EMT, which could be the origin of FAP, according to Zeisberg et al. Therefor, prior studies might not have been as effective as expected is because they did not fully consider cellular localisation differences in FAP expression. Further study into inhibition of FAP should attache great importance to tumor cells of FAP overexpression instead of only Mesenchymal cells. See figure 3 A-F. | 4-5.7 |
| **DISCUSSION** | | |  |
| Summary of evidence | 24 | Several important implications: **First**, FAP expression positively correlated with poor survival and high risks of tumour invasions, lymph node metastases and distant metastases, which shows that this protein may be a promising therapeutic target. **Second**, further [stratification](app:ds:stratification) [analysis](app:ds:analysis) indicated that the patients with overexpression of FAP in tumor cells had a worse outcome and higher risk of tumour metastasis than those with only stroma expression. EMT is recognised as one potential mechanism of it. Therefor, prior studies might not have been as effective as expected is because they did not fully consider cellular localisation differences in FAP expression. Further study into inhibition of FAP should attache great importance to tumor cells of FAP overexpression instead of only Mesenchymal cells. **Third**, fortunately, in this meta-analysis, poor outcomes correlated with higher risks of tumour invasions, lymph node metastasis and distant metastases in patients with high FAP expressions, which demonstrated the accuracy of FAP as a predictive factor. | 7-8 |
| Limitations | 25 | several limitations of this meta-analysis should be noted. First, because this study is a literature-based analysis regarding different types of tumors, the potential for publication bias exists because positive results were predominantly published, inflating this study’s final estimate. Second, differences in including tumors affecting different organs, several characteristics of the study designs, and the inclusion of patients whose treatment included adjuvant therapy may have caused wide heterogeneity in the results among the included studies. Stratified analysis of each analysis characteristic corresponding to a tumor type or treatment-related factors would be helpful to reduce the heterogeneity and to improve the quality of the meta-analysis. However, limited studies provided information concerning FAP expression by subgroups; thus, such analyses are impossible. Finally, the role of FAP interactions with the tumor environment [51, 52] was not assessed in this analysis because the original data from the selected studies did not contain such information. | 8 |
| Conclusions | 26 | In conclusion, this meta-analysis indicated that patients with FAP overexpression in solid tumors have a higher risk of cancer lymph node metastasis and worse prognosis than patients with low FAP expression. The influence of FAP may be greater in those patients with FAP overexpression in tumor cells. The association between FAP overexpression and a detrimental OS in colorectal cancers and pancreatic cancers is similar to that observed for pooled non-colorectal cancers and non-pancreatic cancers, respectively. This analysis suggests that FAP may be a promising therapeutic approach for developing strategies against this protein, not only aiming at its interstitial expression in the tumor microenvironment but also in tumor cells. Further research must be conducted to ascertain the accuracy of the analysis data from this study regarding tumor types or by further prospective studies with larger sample sizes. | 8 |
| **FUNDING** | | |  |
| Funding | 27 | The authors have no support or funding. |  |

**Table S1.** [**PRISMA (Preferred Reporting Items for Systematic Reviews and Meta-Analyses) checklist**](http://www.prisma-statement.org/statement.htm)**.**

Page 2 of 2
